# Accurate Reconstruction of Insertion-Deletion Histories by Statistical Phylogenetics

**DOI:** 10.1371/journal.pone.0034572

**Published:** 2012-04-20

**Authors:** Oscar Westesson, Gerton Lunter, Benedict Paten, Ian Holmes

**Affiliations:** 1 University of California Berkeley and University of California San Francisco Graduate Program in Bioengineering, University of California, Berkeley, California, United States of America; 2 Wellcome Trust Center for Human Genetics, Oxford, Oxford, United Kingdom; 3 Baskin School of Engineering, University of California Santa Cruz, Santa Cruz, California, United States of America; British Columbia Centre for Excellence in HIV/AIDS, Canada

## Abstract

The Multiple Sequence Alignment (MSA) is a computational abstraction that represents a partial summary either of indel history, or of structural similarity. Taking the former view (indel history), it is possible to use formal automata theory to generalize the phylogenetic likelihood framework for finite substitution models (Dayhoff's probability matrices and Felsenstein's pruning algorithm) to arbitrary-length sequences. In this paper, we report results of a simulation-based benchmark of several methods for reconstruction of indel history. The methods tested include a relatively new algorithm for statistical marginalization of MSAs that sums over a stochastically-sampled ensemble of the most probable evolutionary histories. For mammalian evolutionary parameters on several different trees, the single most likely history sampled by our algorithm appears less biased than histories reconstructed by other MSA methods. The algorithm can also be used for alignment-free inference, where the MSA is explicitly summed out of the analysis. As an illustration of our method, we discuss reconstruction of the evolutionary histories of human protein-coding genes.

## Introduction

The Multiple Sequence Alignment (MSA), indispensable to computational sequence analysis, represents a hypothetical claim about the homology beteen sequences. MSAs have many different uses, but the underlying hypothesis can often be classified as a claim either of *structural* homology (the 3D structures align in a particular way) or of *evolutionary* homology (the sequences are related by a particular history on a given phylogenetic tree). These types of hypothesis are similar, but with subtle (and important) distinctions: at the residue level, a claim of evolutionary homology (direct shared descent) is far stronger than a claim of structural homology (same approximate fold). Furthermore, both types of MSA–evolutionary and structural–typically only represent *summaries* of the respective homologies: some fine detail is often omitted. For example, an evolutionary MSA may–or may not–include the ancestral sequences at internal nodes of the underlying tree.

Structural and evolutionary MSAs are often conflated, but they have quite different applications. For example, a common use for a structural MSA is *template-based structure prediction*, where a query sequence is aligned to a target of known structure; the success of this prediction reflects the number of query-template residues correctly aligned [1]. By way of contrast, a common application for an evolutionary MSA is to identify regions or sites under selection, the success of which depends on accurate reconstruction of the evolutionary history [2], [3].

Evaluation of alignment methods is typically done with implicit regard for the structural interpretation. Many benchmarks have used metrics based on the Sum of Pairs Score (SPS) [4]. In the situation that a query-template pairwise alignment is randomly picked out of the MSA, the SPS effectively estimates the proportion of homologous residues that are correctly identified. Several alignment methods attempt to maximize the posterior expectation of SPS or similar metrics. This appears to improve accuracy, particularly when measured with reference to structural homology. However, it does not automatically confer *evolutionary* accuracy – a correct reconstruction of the evolutionary history of the sequences.

Several studies suggest that multiple alignment for evolutionary purposes is still a highly uncertain procedure [5], and that errors therein may significantly bias analyses of evolutionary effects [6]–[11]. A useful component of these studies is simulation of genetic sequence evolution [6], which appears to better indicate evolutionary accuracy than benchmarks derived from protein structure alignments. Simulations can be made quite realistic given the abundance of comparative sequence data [12].

The current state-of-the-art in phylogenetic alignment software is a choice between (on the one hand) programs that lack explicit models of the underlying evolutionary process, and so are not framed as statistical inference problems [6], and (on the other hand) Bayesian Markov chain Monte Carlo (MCMC) methods, which are statistically exact but prohibitively slow [13],[14].

**Figure 1:**
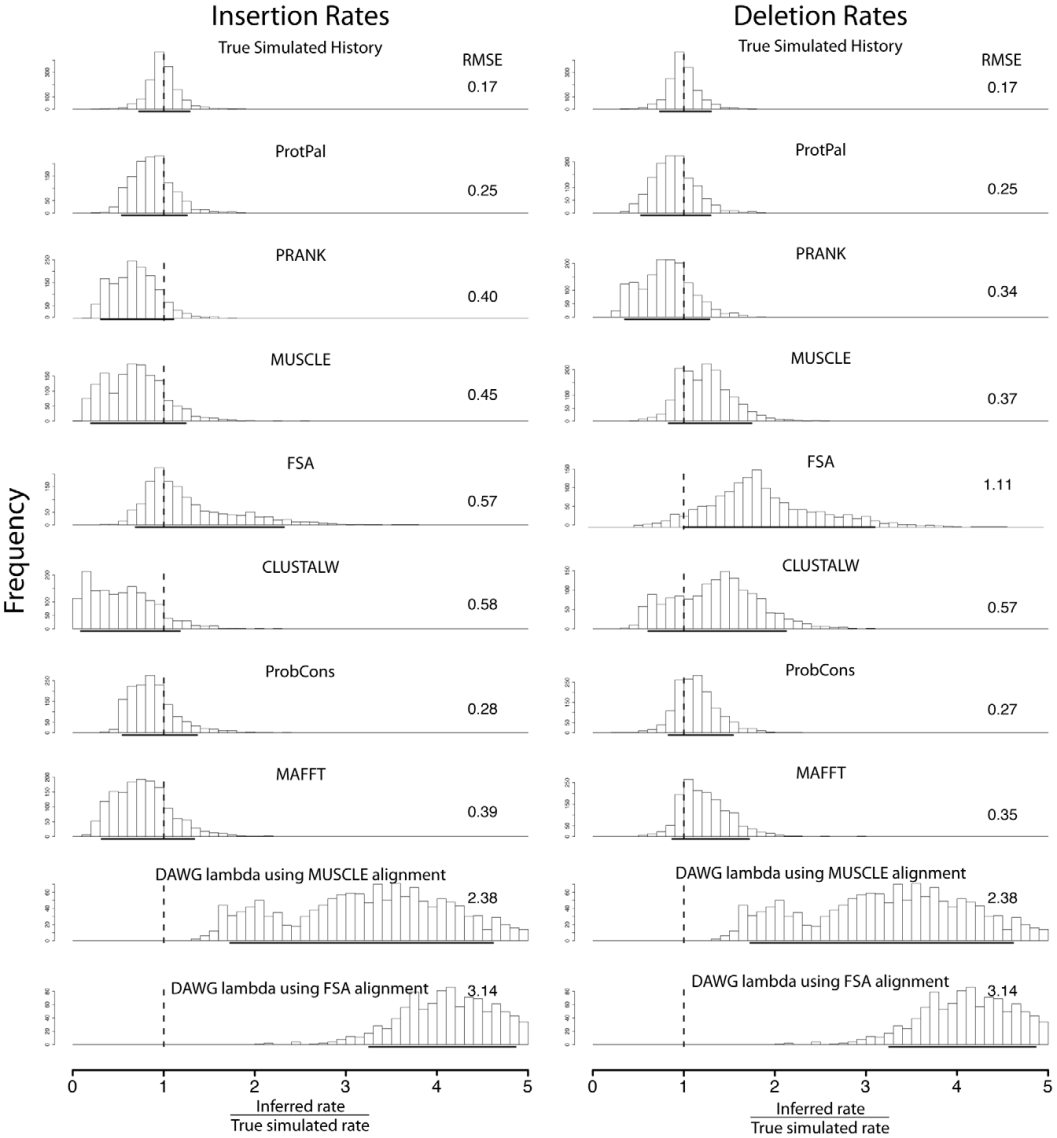
ProtPal's estimates of insertion and deletion rates are the most accurate of any program tested, as measured by the RMSE of 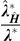 values aggregated over all substitution/indel rate categories. Quantiles containing 90% of the data are shown as a bolded portion of the 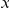-axis, and RMSE is shown to the right of each distribution, the latter computed as described in 1 Equation 1. No aligner approaches the accuracy of the rates estimated with the true alignment, though ProtPal, PRANK, and ProbCons are the top three, with ProtPal as the most accurate over all. Many aligners, particularly MUSCLE, CLUSTALW, and MAFFT, significantly underestimate insertion rates and overestimate deletion rates. ProtPal and PRANK perform their own ancestral reconstruction and other alignment programs were augmented with a most-recent-common-ancestor (MRCA) parsimony as described in [55].

**Figure 2:**
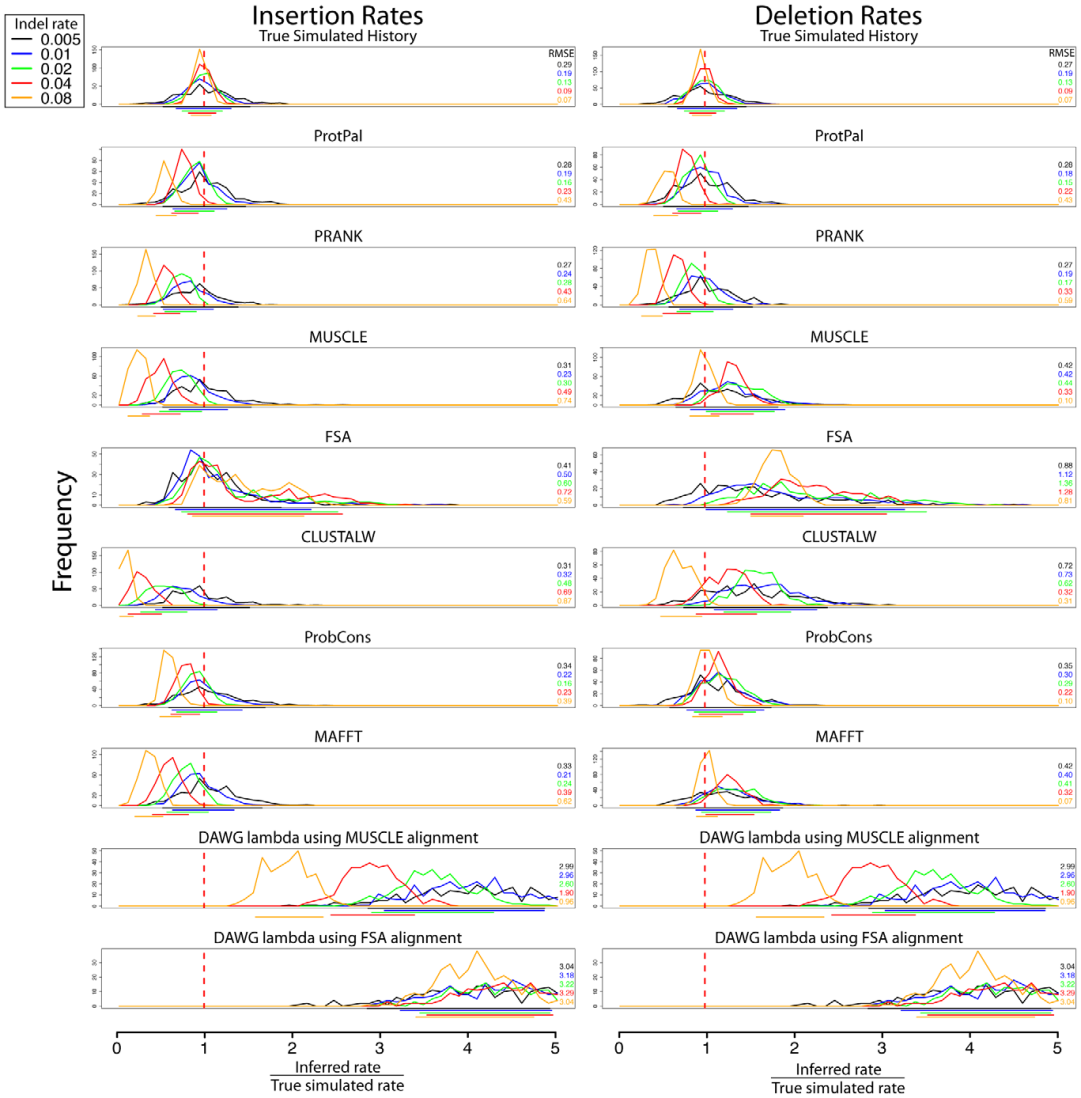
Rate estimation accuracy is highly dependent on the simulated indel rate. For instance, PRANK is more accurate at lower indel rates, ProbCons is more accurate at higher rates. ProtPal is more accurate than PRANK in all but one rate (0.005) and equal or more accurate than ProbCons in all but one rate (0.08). The drift towards 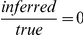 exhibited by most programs indicates that most programs infer proportionally fewer indels as rates are increased, likely due to various forms of gap attraction. Color-coded 90% quantiles and RMSEs are shown underneath and to the right of each group of distributions, respectively. RMSE is computed as described in 1 Equation 1.

A telling observation is that while substitution rate is routinely measured from MSAs and used as an indicator of natural selection, there is relatively little analogous use of indel rate. As we report here, it seems highly likely that even if indel rate is a useful evolutionary signal (which is eminently plausible), the present alignment methods distort measurements of this rate so far as to make it meaningless (Figure 1 and Figure 2).

In this paper, we frame phylogenetic sequence alignment as an approximate maximum likelihood (ML) inference. Our inference algorithm assumes that the tree is known, requiring a separate tree estimation protocol. While this is a strong assumption, it is in principle shared among all progressive aligners (e.g. PRANK [15], Muscle [16], ClustalW [17], MAFFT [18]). The alignment-marginalized likelihoods reported by our algorithm allow for statistical tests between alternative trees, and the functionality to estimate an initial alignment and guide tree from unaligned sequences exists elsewhere in the DART package. Our framing uses automata-theoretic methods from computational linguistics to unify several previously-disjoint areas of bioinformatics: Felsenstein's pruning algorithm for the phylogenetic likelihood function [19], progressive multiple sequence alignment [20], and alignment ensemble representation using partial order graphs [21]. Our algorithm may be viewed as a stochastic generalization of pruning to infinite state spaces: it retains the linear time and memory complexity of pruning (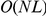 for 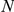 sequences of length 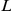), while moderating the biasing effect of the MSA. The algorithmic details of our method are outlined briefly in the Methods, and in more complete, mathematically precise terms (with a tutorial introduction) in a separately submitted work.

Our software implementation of this algorithm is called ProtPal. We measured the accuracy of ProtPal relative to leading non-MCMC alignment/reconstruction protocols by simulating indels and substitutions on a known phylogeny, withholding the true history and attempting to reconstruct it from the sequences at the tips of the tree. The results show that all previous approaches to the reconstruction of ancestral sequences introduce significant biases, including systematic underestimation of insertions and overestimation of deletions. This contradicts previous claims that advances in the statistical foundations of alignment tools, supported by improvements in protein-structure benchmarks, necessarily improve the accuracy of evolutionary parameter estimates like the indel rate [6], [22], [23].

**Figure 3:**
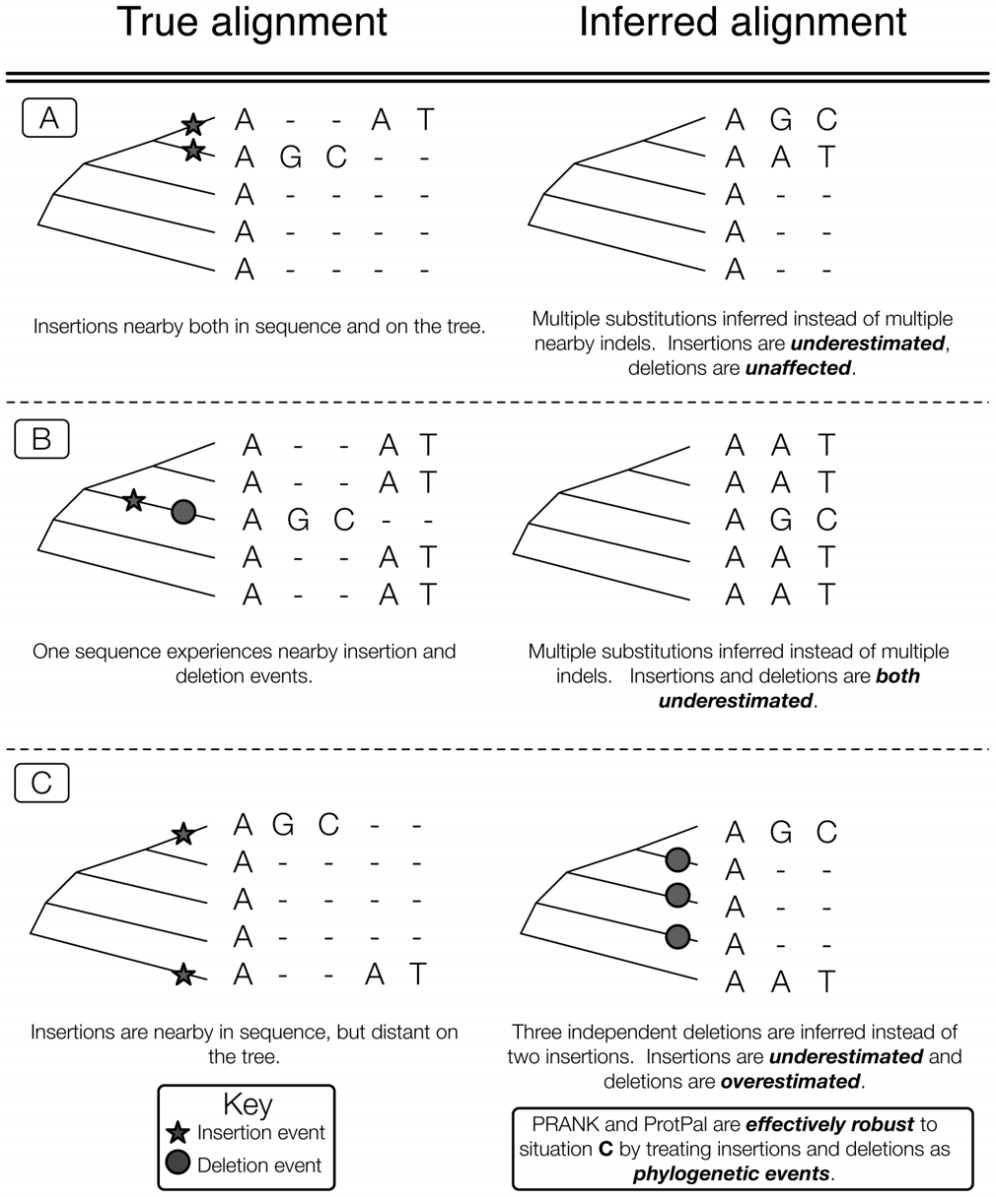
*Gap attraction*, the canceling of nearby complementary indels, can affect insertion and deletion rates in various ways depending on the phylogenetic relationship of the sequences involved. All programs are, to some extent, sensitive to situations **A** and **B** whereas phylogenetic aligners can avoid situation **C**. An insertion at a leaf requires gaps at all other leaves - an understandably costly alignment move when gaps are added without regard to the phylogeny, resulting in **multiple penalization** for each insertion. Such a penalization would cause most non-phylogenetic aligners to prefer the “Inferred alignment” in case **C** where there are fewer total gaps. Aligners treating indels as phylogenetic events would penalize each of the implied multiple deletions and only penalize each insertion once, thus preferring the “True alignment” in case **C**.

ProtPal introduces less bias than any other methods we tested, including PRANK, the state-of-the-art phylogenetic progressive aligner [6]. Both PRANK and ProtPal treat insertions and deletions as phylogenetic events (Figure 3). Based on our tests, ProtPal appears to be the best choice for small to moderately-sized analyses, such as a reconstruction of the history of proteins at the inter-species level in human evolutionary history. Using ProtPal to estimate indel rates for 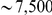 human protein-coding gene families, we find that per-gene indel rates are approximately gamma-distributed, with 95% of genes experiencing a mean rate of less than 0.1 indel events per synonymous substitution event. We find that lengths of inserted and deleted sequences are comparably distributed, having medians 5 and 7, respectively. The human lineage appears to have experienced unusually many insertions since the human-mouse split. By mapping genes to Gene Ontology (GO) terms, we find that the 200 fastest-indel genes are enriched for regulatory and metabolic functions. Possible applications and extensions of our algorithm include phylogenetic placement, homology detection, and reconstruction of structured RNA.

## Results

### Computational reconstruction of simulated histories

We undertook to determine the ability of leading bioinformatics programs, including ProtPal, to characterize mutation event histories. We simulated indel histories on a tree, then attempted to reconstruct the MAP history, 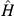, using only knowledge of the sequences 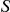 and the phylogeny 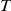 (but not the sequence alignment). The history 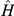 is the aligned set of observed extant and predicted ancestral sequences, such that insertion, deletion, and substitution events can be pinpointed to specific tree branches (though not to specific time points on those branches).

We then characterized the reconstruction quality both directly, by comparison of 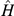 to the true 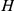, and indirectly, by using 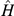 to estimate 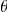, the evolutionary parameters:
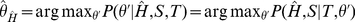
(1)where the latter step assumes a flat prior, 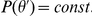 We then compared the history-conditioned parameter estimate 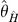 to the true 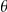.

This statistic is not without its problems. For one thing, we use an initial guess of 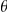 to estimate 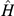. Furthermore, for an unbiased estimate, we should sum over all histories, rather than conditioning on the MAP reconstructed history. This summing over histories would, however, require multiple expensive calculations of 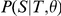, where conditioning on 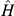 requires only one such calculation. Furthermore, parameter estimation conditioned on a MAP-reconstructed history is the *de facto* method employed by large-scale genomics studies focusing on indels [24]–[27].

#### Simulation model parameters

The model parameters are 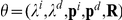: the insertion and deletion rates (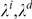), indel length distributions (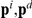) and substitution rate matrix (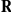). Here we focus on the rates (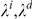).

**Figure 4:**
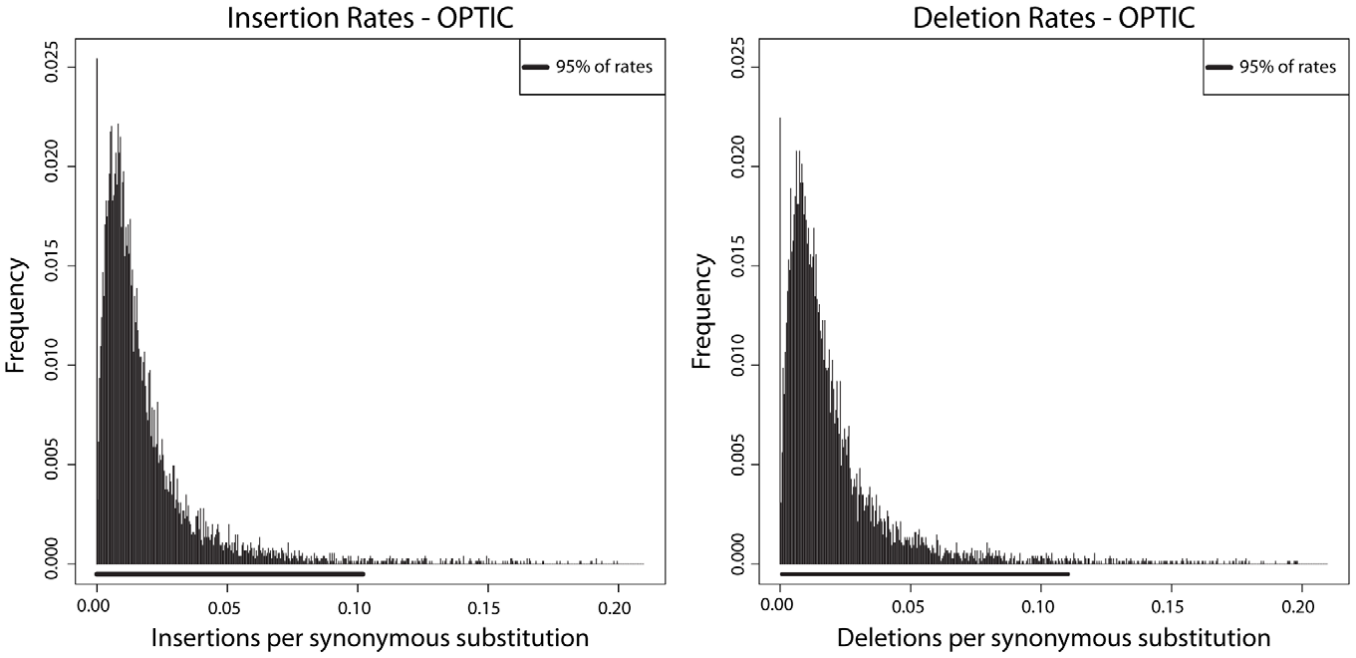
Insertion and deletion rates in *Amniota* show similar distributions, with 95% of genes having rates less than approximately 0.1 indels per synonymous substitution. Insertion and deletion rates were estimated using reconstructions done with ProtPal from a set of approximately 7,500 protein-coding genes from the OPTIC amniote database [30]. Indel rates were normalized by the synonymous substitution rate of each gene as computed with PAML [53] so that the plotted rate represents the number of expected indels per synonymous substitution. Since these rates are conditioned on the MAP reconstructed history, there are many alignments whose inferred indel rates are zero (197, 174, and 54 for insertions, deletions, and both, respectively).

As described in Text S1, we generated data using an external simulation tool, indel-seq-gen, varying insertion (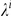), deletion (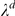) and substitution rates (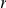) over a range representative of per-gene rates in *Amniota* evolution (Figure 4). We varied indel rates (with 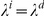) between 0.005 and 0.08 expected indels per unit time, estimating that this range accounts for 95% of human gene families. We left the substitution model 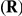 and indel length distributions 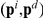 fixed, employing indel-seq-gen's empirically-estimated values.

We performed simulations on mammalian, amniote and fruitfly phylogenies, using the taxa in those clades for which genomic sequence is actually available. We found generally consistent results, with common trends that were most pronounced on the largest of the three trees that we used (the twelve sequenced *Drosophila* species [28]). In discussing the trends, we will refer specifically to the results on this largest of the trees.

### Indel rate estimates

#### Overall most accurate

We first set out to determine which program, when used to analyze a set of unaligned sequences, returns the indel rate estimate closest to the true rate.

We report the ratio of inferred rate to true rate for insertions 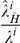 and deletions 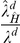 in Figure 1, with each 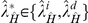 defined as 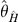 in Equation 1. No parameter estimate derived from a computationally reconstructed history approaches the level of accuracy achieved using the true history (labeled “True simulated history” in Figure 1).

The results do not always concord with previous benchmarks that have measured accuracy using 3D structural alignments: for example, the FSA program, one of the most accurate aligners on structural benchmarks [23], performs poorly here. This discordance may be due to the fundamental differences between evolutionary and structural homology, and the metrics used to assess each. For instance, consider a region with many nearby and overlapping insertions and deletions. The spatial and temporal location of these insertion and deletion events (in particular, the pinpointing of events to branches on the tree) defines what the “perfect” evolutionary reconstruction is. In contrast, even given perfect knowledge of the insertion/deletion history, a “perfect” structural alignment depends only on the proteins at the tips of the tree, and this alignment could differ from the true evolutionary reconstruction.

Fundamentally, the difference between FSA and ProtPal is the underlying metric that is being optimized by each program: FSA attempts to maximize a metric (AMA = Alignment Metric Accuracy) which is essentially “structural” (in the sense that it predicts how many residues would be correctly aligned in a pairwise alignment of two leaf-node sequences, as might be used in structure prediction by target-template alignment), while ProtPal attempts to maximize a “phylogenetic” metric (the probability of a given evolutionary history). The metric we have used in our benchmark (which counts correct reconstruction of the number of indel events on branches of the tree) is also “phylogenetic”. When ranking the programs using the AMA metric, FSA perfoms well, with accuracy exceeding that of ProtPal in the highest indel rate category (Text S1). This suggests that the differences between our evolutionary benchmark and previous benchmarks are not due to the data, but rather the types of metrics that are used to measure alignment accuracy; similarly, the differences between the leading programs are primarily due to what types of benchmark they are explicitly trying to perform well at.

All programs other than ProtPal display insertion- *versus*-deletion biases that are, to a varying degree, asymmetric. Typically, the asymmetry is that insertions are underrepresented and deletions overrepresented. ProtPal's bias, which is generally less than the other programs, is also the most symmetric: reconstructed insertions and deletions are roughly equally reliable, with both slightly underestimated. Over the distribution of human gene rates used by this benchmark, our phylogenetic likelihood approach, ProtPal, provides the most accurate reconstructions of both insertion and deletion counts. PRANK, which also uses a tree (but no likelihood), avoids insertion-deletion biases to a certain extent, although insertion rates are slightly underestimated relative to deletion rates. Since ProtPal's MAP history estimation appears similar to the optimization algorithm of PRANK, we suspect that ProtPal's marginally better performance is due primarily to its main difference in implementation: ProtPal tracks an *ensemble* of possible reconstructions during progressive tree traversal (Section), whereas PRANK uses a single “current best guess.”

#### Effect of indel rate variation

To investigate the effect of indel rate variation on estimation accuracy, we separate each program's error distributions by indel rate (Figure 2). We find that all programs' accuracy is strongly affected by the indel rate used in simulation. As the true indel rate increases, most programs' estimates drift towards 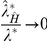. This is consistent with the so-called “gap attraction” effect, where indels that are nearby in sequence can be misinterpreted as substitution events [29]. Depending on the phylogenetic orientation of the events, estimated rates can be elevated or lowered, with different biases for insertion and deletion rates (Figure 3).

Gap attraction and other biases operate simultaneously, and are sometimes opposed. MUSCLE over-estimates the deletion rate under most conditions, but (consistent with a trend where programs have lower 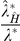 at higher indel rates) gets the deletion rate roughly correct in the highest-indel-rate category of our benchmark. However, the alignments produced by MUSCLE at high indel rates are no more “accurate” by pairwise metrics (Text S1). We conjecture that multiple, contradictory types of gap attraction are at work, e.g. Figures 3B and 3C.

After ProtPal, the two most accurate reconstruction methods are PRANK and ProbCons (the latter combined with a parsimonious indel reconstruction). ProbCons produces more reliable insertion estimates than PRANK in a broad range of benchmark categories, is tied with PRANK for deletion estimates, and appears robust to indel rate variation. PRANK performs slightly better than ProbCons in the slowest indel rate category we considered. ProtPal produces the most reliable estimates overall, outperforming ProbCons in all but the fastest indel rate category, and PRANK in all but the slowest.

#### Sensitivity to substitution rate

As compared to variation of simulated indel rate, variation of simulated substitution rate appears to have little effect on the accuracy of indel reconstruction (Text S1). One notable exception is FSA, which appears to be affected by the substitution rate more than the other programs. For example, when the simulated indel and substitution rates are both low, FSA is comparable to the most accurate of the other programs (ProtPal); but when the substitution rate is increased, FSA's error is greater than the least accurate program (CLUSTALW). Errors in estimating the substitution rate are comparable among the programs tested, and are similarly correlated with the simulation indel rate (Text S1).

### Reconstructed indel histories of human genes

We present here a comprehensive set of reconstructions accounting for the evolutionary history of individual codons in human genes. We used genes in the **O**rthologous and **P**aralogous **T**ranscripts in **C**lades (OPTIC) database's *Amniota* set, comprised of the 5 mammals H. *sapiens*, M. *musculus*, C. *familiaris*, M. *domestica*, O. *anatinus* and G. *gallus* as an outgroup [30]. Considering only those families with one unique ortholog per species (approximately 7,500 families), we combined tree branch statistics across genes, using the species tree in Text S1. Our reconstructions are available at http://biowiki.org/oscar/optic_reconstruction.tar, and we provide here various graphical summaries of *Amniota* evolutionary history. Several negative results stand in contrast to earlier-reported trends.

#### Indel rates

Insertion and deletion rates are approximately gamma-distributed (Figure 4). Roughly 95% of genes have indel rates 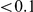 indels per synonymous substitution.

#### Phylogenetic origins

In our simulations, ProtPal pinpoints residues' “branch of origin” more reliably than other tools, with a 93% accuracy rate (Text S1). Many codons appeared to have been inserted following the human-mouse split (Text S1)

#### Branch-specific indel rates

Using our reconstructions to estimate the rates of indel mutations along specific tree branches, we find evidence of an elevated insertion rate in the human (black) branch, as well as on the the *Amniota - Australophenids* (pink) branch (Text S1).

#### Amino acid distributions

Distributions over amino acids differ significantly between inserted, deleted and non-indel sequences (Text S1). In general, small residues are over-represented in insertions, in agreement with previous studies [31].

#### Indel lengths

We find, contrary to a previous study in *Nematode* [32], that length distributions in the Amniotes are nearly identical between insertions and deletions (Text S1). The previously-reported result may be attributable to the deletion-biased nature of the methods used, particularly CLUSTALW and MUSCLE [32].

#### Indel position

The position of indels within genes is highly biased towards the ends of genes, presumably in large part reflecting annotation error (Text S1). The bias is strongest for deletions at the N-terminus of the gene, but both insertions and deletions are enriched in both C- and N- termini.

#### Evolutionary context of indel SNPs

We find no general correlation between the indel rate for a gene and the number of indel polymorphisms recorded for that gene in dbSNP [33] (Text S1).

#### Gene ontology indel rates

No Gene Ontology (GO) categories stand out as having significantly lowered or heightened indel rates in any of the three ontologies, contrasting with the reported results of a 2007 study using a smaller number of genes [31]. An enrichment analysis conducted with GOstat [34] showed that the 200 fastest evolving genes in our data are significantly enriched for regulatory and metabolic functions.

## Discussion

We developed and analyzed a simulation benchmark that compares programs based on their reconstructions of evolutionary history, using instantaneous mutation rates representative of Amniote evolution. We tested several different tree topologies; results were similar on all trees, but most pronounced on the tree with the longest branch lengths. We find that most programs distort indel rate measurements, despite claims to the contrary. Moreover, the systematic bias varies significantly when the rates of substitutions and indels are varied within a biologically reasonable range. Many of the programs we rated have been ranked in the past, but using benchmarks that use protein structural alignments as a gold standard, rather than evolutionary simulations. Furthermore, these previous benchmarks have not directly assessed the reconstruction of evolutionary history (or summary statistics such as the indel rate), but have used other alignment accuracy metrics such as the *Sum of Pairs Score*. Alignment programs that perform weakly on our benchmark have apparently performed well on these previous benchmarks. We hypothesize that these benchmarks, compared to ours, are less directly predictive of a program's accuracy at historical reconstruction, although they may better reflect the program's suitability to assist in tasks relating more closely to folded structure, like prediction of a protein's 3D structure from a homologous template.

We have introduced a new notation that describes a general, hidden Markov model-structured likelihood function for indel histories on a tree, as well as the structure of the corresponding inference algorithm. We have implemented the new method in a freely-available program, ProtPal, that allows, for the first time, phylogenetic reconstruction with accuracy over a broad range of indel rates. ProtPal is written in C++ as a part of the DART package: www.biowiki.org/ProtPal. The evolutionary reconstructions ProtPal produces are, according to our simulated tests, the most accurate of any available tool, for a range of parameters typical of human genes.

We applied ProtPal to the reconstruction of human gene indel history, using families of human gene orthologs from the OPTIC database. We find some patterns that agree with previous studies, such as the non-uniform distributions over amino acids seen in [31]. Other results stand in contrast - a previous study found significantly different length distributions for insertions and deletions [32], whereas in our data they appear very similar. Another prediction of our reconstruction is an elevated rate of insertions on the human branch since the human-mouse split. This contrasts with a previous analysis [35], though the data therein was whole genomes, rather than individual protein-coding genes. In contrast to [31], we find no obvious predictive power of the Gene Ontology (GO) for indel rates; that is, the indel rate does not appear strongly correlated with the presence or absence of any particular GO term-gene association. However, enrichment analysis for GO terms using GOstat [34] showed that the 200 fastest-evolving genes are significantly enriched for regulatory and metabolic function. This apparent discrepancy might be explained by a group of regulatory and metabolic genes which have very high indel rates, but whose small number prevent them from skewing the average within their GO categories.

Many applications which use a fixed-alignment phylogenetic likelihood could potentially benefit from ProtPal's reconstruction profiles. For example, phylogenetic placement algorithms estimate taxonomic distributions by evaluating the relative likelihoods of placing sequence reads on tree branches [36]. By using sequence profiles exported from ProtPal, these reads could be placed with greater attention to indels and a more realistic accounting for alignment uncertainty. Homology detection could be done in a similar way, thereby making use of the phylogenetic relationship of the sequences within the reference family. It has been observed that the detection of positive selection is highly sensitive to the alignment used [7]. ProtPal could be modified to detect selection using entire profiles rather than single alignments, potentially eliminating the bias brought on by an inaccurate alignment.

In summary, multiple alignments are frequently constructed for use in downstream evolutionary analyses. However, except for our method and slow-performing MCMC methods, there are no software tools for reconstructing molecular evolutionary history that explicitly maximize a phylogenetic likelihood for indels. Our results strongly indicate that algorithms such as ProtPal (which use such a phylogenetic model) produce significantly more reliable estimates of evolutionary parameters, which we believe to be highly indicative of evolutionary accuracy. These results falsify previous assertions that existing, non-phylogenetic tools are well-suited to this purpose. Furthermore, we have demonstrated that it is possible to achieve such accuracy without sacrificing asymptotic guarantees on time/memory complexity, or resorting to expensive MCMC methods. ProtPal can reconstruct phylogenetic histories of entire databases on commodity hardware, enabling the large-scale study of evolutionary history in a consistent phylogenetic framework.

## Methods

The details concerning generation and analysis of simulated data are contained in Text S1. A mathematically complete description of the alignment algorithm has been submitted as a separate work, and an early version has been made available online here: http://arxiv.org/abs/1103.4347.

### Felsenstein's algorithm for indel models

Our algorithm may be viewed as a generalization of Felsenstein's pruning recursion [19], a widely-used algorithm in bioinformatics and molecular evolution. A few common applications of this algorithm include estimation of substitution rates [37]; reconstruction of phylogenetic trees [38]; identification of conserved (slow-evolving) or recently-adapted (fast-evolving) elements in proteins and DNA [39]; detection of different substitution matrix “signatures” (e.g. purifying vs diversifying selection at synonymous codon positions [40], hydrophobic vs hydrophilic amino acid signatures [41], CpG methylation in genomes [42], or basepair covariation in RNA structures [43]); annotation of structures in genomes [44], [45]; and placement of metagenomic reads on phylogenetic trees [36].

Felsenstein's algorithm computes 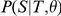 for a substitution model by tabulating intermediate probability functions of the form 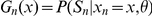, where 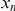 represents the individual residue state of ancestral node 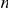, and 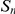 represents all the sequence data that is causally descended from node 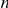 in the tree (i.e. the observed residues at the set of leaf nodes whose most recent common ancestor is node 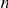).

The pruning recursion visits all nodes in postorder. Each 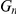 function is computed in terms of the functions 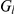 and 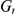 of its immediate left and right children (assuming a binary tree):
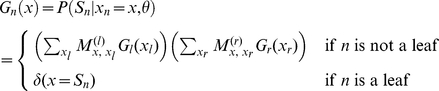
where 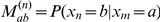 is the probability that node 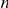 has state 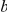, given that its parent node 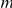 has state 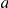; and 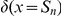 is a Kronecker delta function terminating the recursion at the leaf nodes of the tree. These 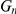 functions are often referred to as “messages” in the machine-learning literature [46].

Our new algorithm is algebraically equivalent to Felsenstein's algorithm, if the concept of a “substitution matrix” over a particular alphabet is extended to the countably-infinite set of all sequences over that alphabet. Our chosen class of “infinite substitution matrix” is one that has a finite representation: namely, the *finite-state transducer*, a probabilistic automaton that transforms an input sequence to an output sequence, and a familiar tool of statistical linguistics [47].

By generalizing the idea of matrix multiplication (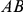) to two transducers (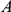 and 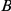), and introducing a notation for feeding the same input sequence to two transducers in parallel (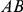), we are able to write Felsenstein's algorithm in a new form (see Section):
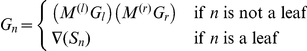
where 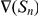 is the transducer equivalent of the Kronecker delta 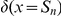. The function 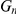 is now encapsulated by a transducer “profile” of node 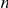.

**Figure 5:**
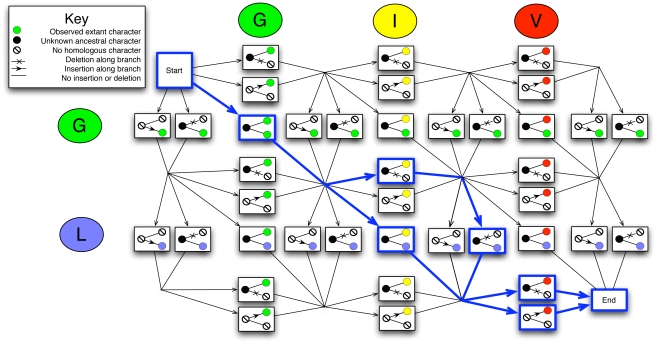
Each path through this state graph represents a possible evolutionary history relating sequences GL and GIV. By using stochastic traceback algorithms (sampling paths proportional to their posterior probability, blue highlighted states and transitions), it is possible to select a high-probability subset of the full state graph. By constructing such a subset at each internal node, it is possible to maintain a bound on the state space size during progressive tree traversal while still retaining an ensemble of possible histories.

This representation has complexity 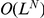 for 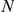 sequences of length 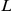, which we reduce to 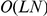 by stochastic approximation of the 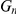. This approximation relies on the *alignment envelope* [48], a data structure introduced by prior work on efficient alignment methods. The alignment envelope is a subset of all the possible histories in which most of the probability mass is concentrated. A related data structure is the *partial order graph* [21]. Both these data structures can be viewed as ensembles of possible histories, in contrast to a single “best-guess” reconstruction of the history. Figure 5 sampledGraph shows a state graph, with paths through it corresponding to histories relating the two sequences GL and GIV. The paths highlighted in blue form a partial order graph, corresponding to a subset of these histories generated by a stochastic traceback. At each progressive traversal step, we sample a high-probability subset of alignments of two sibling profiles in order to maintain a bound on the state space size. Note that if we sample only the most likely path at every internal node, we essentially recover the progressive algorithm of PRANK, and if we sample and store all solutions, we recover the machine 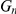 with state space of size 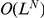.

### Transducer definitions and lemmas

The definitions and lemmas are presented in a condensed form here, and expanded upon in [49].

A transducer is a tuple 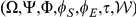 where 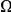 is an input alphabet, 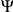 is an output alphabet, 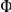 is a set of states, 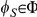 is the start state, 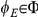 is the end state, 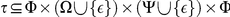 is the transition relation, and 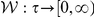 is the transition weight function.

Suppose that 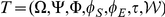 and 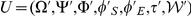 are transducers.

Let 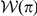 be the product of all transition weights along a state path 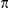 and let 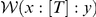 be the sum of such weights for all paths whose input labels, concatenated, yield the string 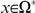 and whose output labels yield 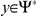.

*Equivalence*: If 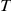 and 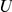 have the same input and output alphabets (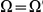 and 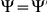) and the same sequence weights 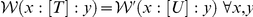, then we say the transducers are *equivalent*, 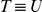. Less formally, we will write 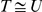 if 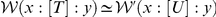.

*Moore transducers*: The *Moore normal form* for transducers, named for Moore machines [50], associates input/output with three distinct types of state: *Match*, *Insert* and *Delete*. Paths through Moore transducers can be associated with (gapped) pairwise alignments of input and output sequences. For any transducer 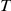, there exists an equivalent Moore-normal form transducer 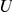 with 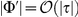 and 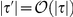.

*Composition*: If 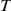
's output alphabet is the same as 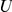
's input alphabet (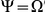), there exists a transducer, 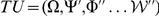, that unifies the output of 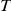 with the input of 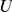, such that 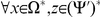:
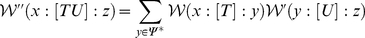
(2)If 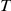 and 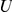 are in Moore form, then 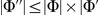 and 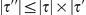.

*Intersection*: If 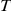 and 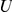 have the same input alphabets (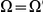), there exists a transducer, 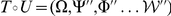, that unifies the input of 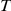 with the input of 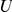. The output alphabet is 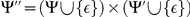, i.e. a 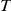-output symbol (or a gap) aligned with a 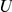-output symbol (or a gap).

Let 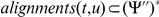 denote the set of all gapped pairwise alignments of sequences 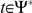 and 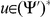. Transducer 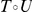 has the property that 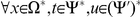:
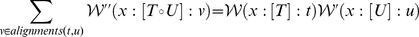
(3)If 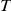 and 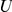 are in Moore form, then 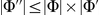 and 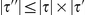. Paths through 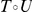 are associated with three-way alignments of the input sequence to the two output sequences.

*Identity*: Let 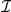 be a transducer that copies input to output unmodified, so 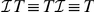.

*Exact match*: For any sequence 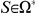, there exists a Moore-form transducer 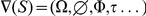 with 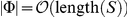 and 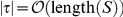, that rejects all input except 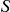, such that 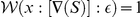 if 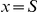, and 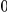 if 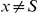. Note that 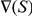 outputs nothing (the empty string).

*Chapman-Kolmogorov transducers*: A transducer 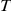 is *probabilistic* if 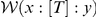 represents a probability 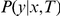: that is, for any given input string, 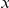, it defines a probability measure on output strings, 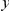.

Suppose 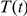 is a function returning a probabilistic transducer of the form 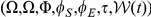, i.e. a transducer whose transition weight 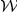 depends on an additional *time parameter*, 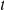, and which satisfies the transducer equivalence 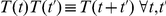.

Then 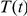 gives the finite-time transition probabilities of a homogeneous continuous-time Markov process on the strings 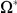, as the above transducer equivalence is a form of the Chapman-Kolmogorov equation.

If the state space of 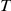 is finite, then this equation describes a renormalization of the composed state space 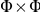 back down to the original state space 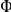. So far, only one nontrivial time-dependent transducer is known that solves this equation exactly using a finite number of states: the TKF91 model [51].

### The phylogenetic likelihood

We rewrite the evidence, 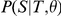 for sequences 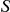, tree 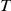, and parameters 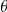, in the form 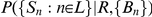 where 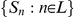 denotes the set of sequences observed at leaf nodes, 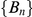 denotes the stochastic evolutionary processes occuring on the branches, and 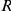 denotes the probabilistic model for the sequence at the root node of the tree.

The root and branch transducers 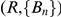 represent an alternative view of the tree and parameters 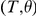. The root transducer 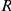 outputs from the equilibrium or other initial distribution of the process. If 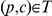 is a parent-child pair, then 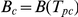 is a time-dependent transducer parameterized by the branch length. In practise, the branch transducers need not satisfy the Chapman-Kolmogorov equation for the following constructs to be of use; for example, the 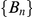 might be approximations to true Chapman-Kolmogorov transducers [52].

Let 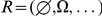 be a transducer outputting sequences sampled from the prior at the phylogenetic root.

Let 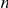 be a tree node. If 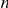 is a leaf, define 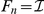. Otherwise, let 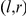 denote the left and right child nodes, and define 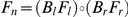 where 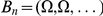 is a transducer modeling the evolution on the branch leading to 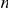.

Diagramatically we can write 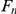 as (.. (.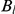. (.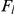.)) (.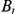. (.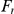.)))

The phylogenetic likelihood is then fully described by 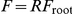.

Like 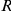, transducer 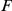 models a probability distribution over output sequences, but accepts only the empty string as an input sequence. This empty input sequence is just a technical formality (transducers must have inputs); if we ignore it, we can think of 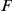 and 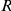 as hidden Markov models (HMMs), rather than transducers. 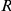 is an HMM that generates a single sequence, 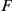 a multi-sequence HMM that generates the whole set of leaf sequences.

Inference with HMMs often uses a dynamic programming matrix (e.g. the Forward matrix) to track the ways that a given evidential sequence can be produced by a given grammar.

For our purposes it is useful to introduce the evidence in a different way, by transforming the model to incorporate the evidence directly. We augment the state space so that the model is no longer capable of generating any sequences *except* the observed 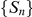, by composing 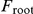
's forked outputs with exact-match transducers that will only accept the observed sequences at the leaves of the tree. This yields a model, 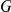, whose state space is of size 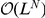 and, in fact, is directly analogous to the Forward matrix.

If 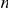 is a leaf node, then let 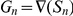 where 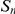 is the sequence at 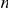. Otherwise, 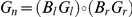.

Diagramatically we can write 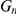 as (.. (.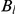..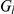.) (.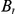. .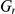.))

Let 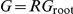. The evidence is 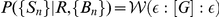.

The net output of 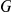 is always the empty string. The sequences 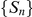 are recognized as inputs by the 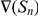 transducers at the tips of the tree, but are not passed on as outputs themselves.

Likewise, the input of 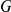 is the empty string, because 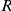 accepts only the empty string on its input.

We can think of 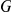 as a Markov model, rather than an HMM. It has no input or output; rather, the sequences are encoded into its structure.

Transducer 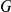 has 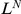 states, which is impractically many, so ProtPal uses a progressive hierarchy 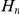 of approximations to the corresponding 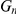, with state spaces that are bounded in size.

If 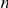 is a leaf node, let 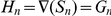. Otherwise, let 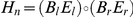 where 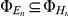 is a subset defined by sampling complete paths through the Markov model 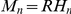 and adding the 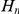-states used by those paths to 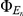, until the pre-specified bound on 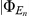 is reached. Then 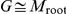.

The likelihood of a given history may be calculated by summing over paths through 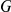 consistent with that history. In the simplest cases (e.g. minimal Moore-form branch transducers), each indel history corresponds to exactly one path, so the MAP indel history corresponds to the maximum-weight state path through 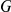.

### Alignment envelopes

Let 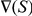 be defined such that it has only one nonzero-weighted path
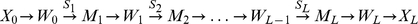
so a 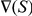-state is either the start state (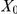), the end state (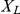), a wait state (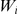) or a match state (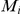). All these states have the form 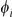 where 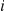 represents the number of symbols of 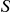 that have to be read in order to reach that state, i.e. a “co-ordinate” into 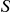. All 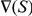-states are labeled with such co-ordinates, as are the states of any transducer that is a composition involving 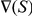, such as 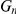 or 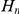.

For example, in a simple case involving a root node (1) with two children (2,3) whose sequences are constrained to be 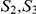, the evidence transducer is 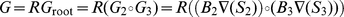 = (.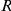. (.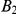..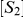.) (.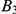..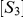.))

All states of 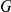 have the form 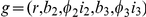 where 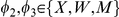, so 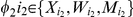 and similarly for 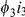. Thus, each state in 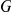 is associated with a co-ordinate pair 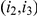 into 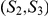, as well as a state-type pair 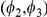.

Let 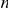 be a node in the tree, let 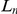 be the set of indices of leaf nodes descended from 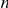, and let 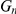 be the phylogenetic transducer for the subtree rooted at 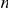, defined in Section. Let 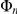 be the state space of 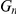.

If 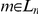 is a leaf node descended from 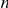, then 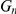 includes, as a component, the transducer 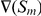. Any 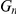-state, 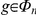, is a tuple, one element of which is a 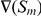-state, 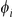, where 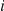 is a co-ordinate (into sequence 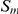) and 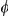 is a state-type. Define 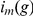 to be the co-ordinate and 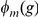 to be the corresponding state-type.

Let 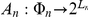 be the function returning the set of *absorbing leaf indices* for a state, such that the existence of a finite-weight transition 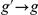 implies that 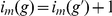 for all 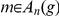.

Let 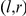 be two sibling nodes. The *alignment envelope* is the set of sibling state-pairs from 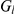 and 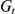 that can be aligned. The function 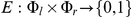 indicates membership of the envelope. For example, this basic envelope allows only sibling co-ordinates separated by a distance 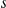 or less
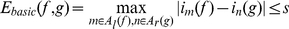
(4)


An alignment envelope can be based on a *guide alignment*. For leaf nodes 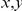 and 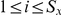, let 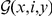 be the number of residues of sequence 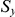 in the section of the guide alignment from the first column, up to and including the column containing residue 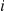 of sequence 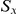.

This envelope excludes a pair of sibling states if they include a homology between residues which is more than 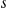 from the homology of those characters contained in the guide alignment:
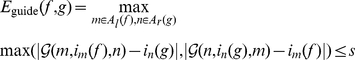
(5)


Let 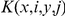 be the number of match columns (those columns of the guide alignment in which both 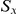 and 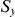 have a non-gap character) between the column containing residue 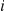 of sequence 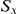 and the column containing residue 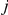 of sequence 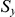. This envelope excludes a pair of sibling states if they include a homology between residues which is more than 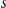 *matches* from the homology of those characters contained in the guide alignment:
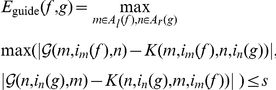



### OPTIC data analysis

#### Data

Amniote gene families were downloaded from http://genserv.anat.ox.ac.uk/downloads/clades/. We restricted our analysis to the 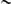
7,500 families having simple 1∶1 orthologies. The same species tree topology (downloaded from http://genserv.anat.ox.ac.uk/clades/amniota/displayPhylogeny was used for all reconstructions, though branch lengths were estimated separately for each family as part of OPTIC. When computing branch-specific indel rates, the branch lengths of the species tree were used.

#### Reconstruction and rate estimation

Gene families were aligned and reconstructed using ProtPal with a 3-rate class Markov chain over amino acids, insertion and deletion rates set to 0.01, and 250 traceback samples. Averaged and per-branch indel rates were computed with ProtPal using the -pi and -pb options. The indel rates were then normalized by the synonymous substitution rate for each corresponding nucleotide alignment (taken directly from OPTIC), computed with PAML [53]. Residues' origins were determined by finding the tree node closest to the root containing a non-gap reconstructed character.

#### External data

Genes were mapped to Gene Ontology terms via the mapping downloaded from http://www.ebi.ac.uk/GOA/human_release.html during 10/2010. Indel SNPs per gene were taken from a table downloaded from Supplemental Table 5 of [54].

## Supporting Information

Text S1 **Contains techinical details concerning generation of simulation data, analysis of OPTIC data, as well as figures pertaining to both simulated and OPTIC data.**
(PDF)
