# Supplementary material for: Accurate Reconstruction of Insertion-Deletion Histories by Statistical Phylogenetics
Source: PLoS One. 2012 Apr 20;7(4):e34572. doi: 10.1371/journal.pone.0034572 (PMC3335033; doi:10.1371/journal.pone.0034572)
Supplement: Text S1 — Contains techinical details concerning generation of simulation data, analysis of OPTIC data, as well as figures pertaining to both simulated and OPTIC data. (PDF) [file pone.0034572.s001.pdf]

Supplemental Information

Oscar Westesson[1], Gerton Lunter[2], Benedict Paten[3], Ian Holmes[1,*]

1 UC Berkeley and UCSF Graduate Program in Bioengineering, University of California, Berkeley, CA, USA;

2 Wellcome Trust Center for Human Genetics, Oxford, Oxford, UK;

3 Baskin School of Engineering, UC Santa Cruz, Santa Cruz, CA, USA

∗ E-mail: protpal@postbox.biowiki.org

1 Methods

1.1 Simulation parameters and setup

Data generation Our simulation study is comprised of alignments simulated using 5 different indel rates (0.005, 0.01, 0.02, 0.04, and 0.08 indels per unit time), each with 3 different substitution rates (0.5, 1, and 2 expected substitutions per unit time) and 100 replicates. Time is defined such that a sequence evolving for time t with substitution rate r is expected to accumulate rt subsitutions per site. We employed an independent third-party simulation program, indel-seq-gen, specifically designed to generate realistic protein evolutionary histories [1]. indel-seq-gen is capable of modeling an empirically-fitted indel length distribution, rate variation among sites, and a neighbor-aware distribution over inserted sequences allowing for small local duplications. Since the indel and substitution model used by indel-seq-gen are separate from (and richer than) those used by ProtPal, ProtPal has no unfair advantage in this test.

indel-seq-gen v2.0.6 was run with the following command:

```
cat guidetree.tree| indel-seq-gen -m JTT -u xia --num_gamma_cats 3 -a
0.372 --branch_scale r/b --outfile simulated_alignment.fa --quiet --outfile_format
f -s 10000 --write_anc
```

The above command uses the "JTT" substition model, the "xia" indel fill model (based on neighbor effects, estimated from E coli k-12 proteins [1]), and 3 gamma-distributed rate categories with shape 0.372. Branch lengths are scaled by the substitution rate for simulation rate r, normalized by the inverse of indel-seq-gen's underlying substitution rate ($b = 1.2$) so as to adhere to the above definition of evolutionary "time". Similarly, indel rates, which are set in the guide tree file `guidetree.tree`, are scaled by $\frac{b}{r}$ so that $t\lambda^*$ insertions/deletions are expected over time t for rate λ^*.

The root mean squared error (RMSE) for each error distribution was computed as follows:

$$RMSE = \sqrt{\sum_{replicates} (\frac{\hat{\lambda}^*_{\hat{H}}}{\lambda^*} - 1)^2} \tag{1}$$

The true tree was made available to all programs which can utilize a tree (ProtPal, PRANK, MUSCLE), representing the use case in which the true tree is known (e.g. via the species tree) but the true alignment is unknown. We ran simulations on three different phylogenies: a tree of twelve sequenced *Drosophila* genomes [2] and trees from the mammalian and amniotic clades of the OPTIC database. We here report results for the *Drosophila* tree, which we empirically observe to show trends consistent with, but more pronounced than, those of the mammalian and amniotic trees. The clearer trends may be due to the *Drosophila* tree being larger than the other trees (12 taxa), or having a diverse range of branch lengths (0.001 - 0.59 expected substitutions/site, at the genome-wide average rate). The simulation data, reconstructions, and analysis scripts are available from `http://biowiki.org/~oscar/protpal_simulation_benchmark.bz2`.

Alignment We investigated several multiple alignment tools [3–8] in combination with alignment-conditioned reconstruction methods. Programs were

run with their default settings, with the exception of PRANK and MUSCLE. To specify ancestral inference, the guide tree, and "insertions opening forever", PRANK used the extra options "-writeanc -t <treefile> +F". PRANK's -F option allows insertions to match characters at alignments closer to the root. This can be a useful heuristic safeguard when an incorrect tree may produce errors in subtree alignments that cannot be corrected at internal nodes closer to the root. Since the true guide tree is provided to PRANK, it is safe to treat insertions in a strict phylogenetic manner via the +F option. For computational efficiency, ProtPal was provided with a CLUSTALW guide alignment. Any alignment of the sequences can be used as a guide, and we chose CLUSTALW for its general poor performance, so that ProtPal would gain no unfair advantage by the information contained in the guide alignment. MUSCLE was provided the guide tree with the additional option "-usetree <treefile>".

Muscle v3.6

```
MUSCLE -in unaligned.fa -out aligned.fa -usetree guidetree.tree
```

PRANK v.080820

```
PRANK -d=unaligned.fa -noxml -realbranches -writeanc -o=output_directory
-t=guidetree.tree +F
```

Clustal v2.03

```
clustalw -INFILE=unaligned.fa -OUTFILE=aligned.fa
```

ProbCons v1.12

```
probcons unaligned.fa > aligned.fa
```

FSA v1.08

```
fsa unaligned.fa > aligned.fa
```

MAFFT v6.818b

```
mafft unaligned > aligned.fa
```

```
muscle 3.6 -in <infile> -out <outfile> -usetree <guide tree>
prank v.
cluswal 2.03 $(clustalw) -INFILE=$< -OUTFILE=$@.clustalw
probcons 1.12 probcons <infile>
fsa 1.08 fsa <infile>
mafft v6.818b mafft <infile>
```

Imputing indel histories The ancestral reconstruction programs ProtPal and PRANK were used to directly impute indel histories. The remaining tools were augmented to reconstruction tools by post-processing their MSAs using the maximum parsimony algorithm described in [9], with the ambiguous cases described therein (e.g. where a column of characters could be equally parsimoniously explained by a deletion on one child branch or an insertion on the other) resolved by a uniformly random choice from the possible solutions. Indel rates were estimated by counting indel events in MAP reconstructed histories:

$$\hat{\theta}_{\hat{H}} = \text{argmax}_{\theta'} P(\theta'|\hat{H}, S, T) = \text{argmax}_{\theta'} P(\hat{H}, S|T, \theta') \quad (2)$$

where the latter step assumes a flat prior, $P(\theta') = \text{const}$.

This statistic is not without its problems. For one thing, we use an initial guess of θ to estimate $\hat{H}$. Furthermore, for an unbiased estimate, we should sum over all histories, rather than conditioning on the MAP reconstructed history. This summing over histories would, however, require multiple expensive calculations of $P(S|T, \theta)$, where conditioning on $\hat{H}$ requires only one such calculation. We further justify our benchmark of parameter estimates conditioned on a MAP-reconstructed history by noting that this the *de facto* method employed by large-scale genomics studies focusing on indels [10–13].

As well as imputing indel rates from reconstructed histories, we also tried

using the `lambda.pl` program in the DAWG package [14], which estimates indel rates from MSAs directly (without attempting reconstruction).

Estimating substitution rates Substitution rates were estimated for each inferred alignment using XRate's built-in EM algorithm and the following simple rate matrix. Given an equilibrium distribution over amino acid characters, with π_i defining the proportion of character i, the rate of character i mutating to j is set to $r\pi_j$ where r is the only free rate parameter. XRate's estimate of r is taken to be the average substitution rate of the MSA.

By using indel-seq-gen's branch-scale option and changing the indel rate parameters accordingly, we are able to modulate the substitution and indel rates independently in the data generation step. This true substitution rate and the rate inferred by XRate are then directly comparable.

2 Additional OPTIC Figures

In addition to estimating indel rates for all genes in the OPTIC set, we performed various other analyses which were left out of the main text for reasons of space limitations. We provide figures those displaying results here.

3 Supplemental Figures

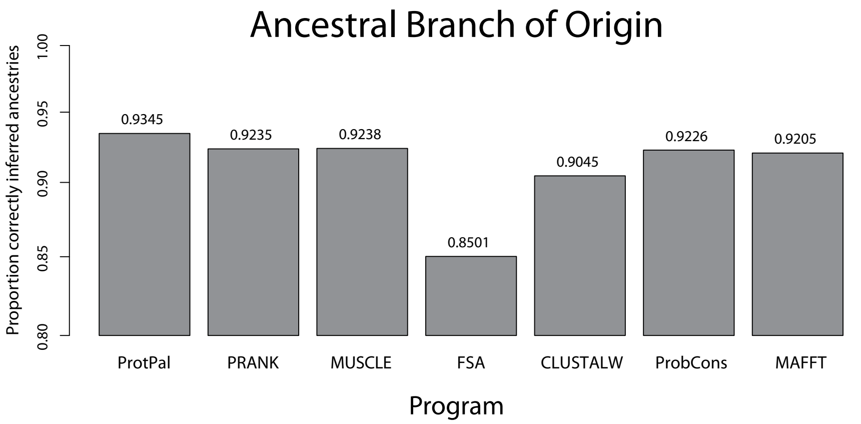

Figure 1: ProtPal correctly reconstructs the age of more extant residues than any other program tested. The y-axis shows the proportion of extant residues whose point of origin on the phylogenetic tree was correctly pinpointed by the reconstruction. The branch of origin was found by taking the tree node closest to the root containing a non-gap reconstructed character. All programs except FSA are in the 92%-94% range, owing to the fact that many columns (especially at low indel rates) are devoid of indels, making inference of origin trivial (as these columns' origin is pre-root).

4 References

References

1. Strope C, Abel K, Scott S, Moriyama E: **Biological sequence simulation for testing complex evolutionary hypotheses: indel-Seq-Gen version 2.0**. *Mol Biol Evol* 2009, **26**(11):2581–93.

2. Clark AG, Eisen MB, Smith DR, Bergman CM, Oliver B, Markow TA, Kaufman TC, Kellis M, Gelbart W, Iyer VN: **Evolution of genes and genomes on the Drosophila phylogeny.** *Nature* 2007, **450**(7167):203–218.

3. Bradley RK, Roberts A, Smoot M, Juvekar S, Do J, Dewey C, Holmes I, Pachter L: **Fast Statistical Alignment**. *PLoS Computational Biology* 2009, **5**(5):e1000392, [http://dx.doi.org/10.1371%2Fjournal.pcbi.1000392].

4. Do CB, Brudno M, Batzoglou S: **PROBCONS: Probabilistic Consistency-based Multiple Alignment of Amino Acid Sequences** 2004. [Submitted.].

5. Edgar RC: **MUSCLE: a multiple sequence alignment method with reduced time and space complexity.** *BMC Bioinformatics* 2004, **5**:113.

6. Katoh K, Kuma K, Toh H, Miyata T: **MAFFT version 5: improvement in accuracy of multiple sequence alignment**. *Nucleic Acids Research* 2005, **33**(2):511–518.

7. Larkin M, Blackshields G, Brown N, Chenna R, McGettigan P, McWilliam H, Valentin F, Wallace I, Wilm A, Lopez R, Thompson J,

Gibson T, Higgins D: **Clustal W and Clustal X version 2.0**. *Bioinformatics* 2007, **23**:2947–2948.

8. Löytynoja A, Goldman N: **An algorithm for progressive multiple alignment of sequences with insertions**. *Proceedings of the National Academy of Sciences of the USA* 2005, **102**(30):10557–62.

9. Sinha S, Siggia E: **Sequence Turnover and Tandem Repeats in cis-Regulatory Modules in Drosophila**. *MBE* 2005, **22**(4).

10. Kamneva O, Liberles A, Ward N: **Genome-Wide Influence of Indel Substitutions on Evolution of Bacteria of the PVC Superphylum, Revealed Using a Novel Computational Method**. *Genome Biology and Evolution* 2010, **2**:870–886.

11. Zhang Z, Huang J, Wang Z, WAng L, Peiji G: **Impact of indels on the flanking regions in structural domains**. *Molecular Biology and Evolution* 2011, **28**:291–301.

12. Zhu L, Wang Q, Tang P, Araki H, Tian D: **Genomewide association between insertions/deletions and the nucleotide diversity in bacteria**. *Molecular Biology and Evolution* 2009, **26**(10):2353–2361.

13. Gomez-Valero L, Latorre A, Gil R, Gadau J, Feldhaar H, Silva F: **Patterns and rates of nucleotide substitution, insertion and deletion in the endosymbiont of ants Blochmannia floridanus**. *Molecular Ecology* 2008, **17**(19):4382–4392.

14. Cartwright RA: **DNA assembly with gaps (Dawg): simulating sequence evolution.** *Bioinformatics* 2005, **21 Suppl 3**:iii31–8.

15. Holmes I: *A DART tutorial.* Berkeley Drosophila Genome Project, LSA Room 539, UC Berkeley 2000. [A tutorial for probabilistic methods and

hidden Markov models, presented with the aid of the author's software package implementing many common HMM algorithms. Available from http://www.fruitfly.org/~ihh/].

16. Wang Z, Martin J, Abubucker S, Yin Y, Gasser R, Mitreva M: **Systematic analysis of insertions and deletions specific to nematode proteins and their proposed functional and evolutionary relevance.** *BMC Evol Biol.* 2009, **9**(23).

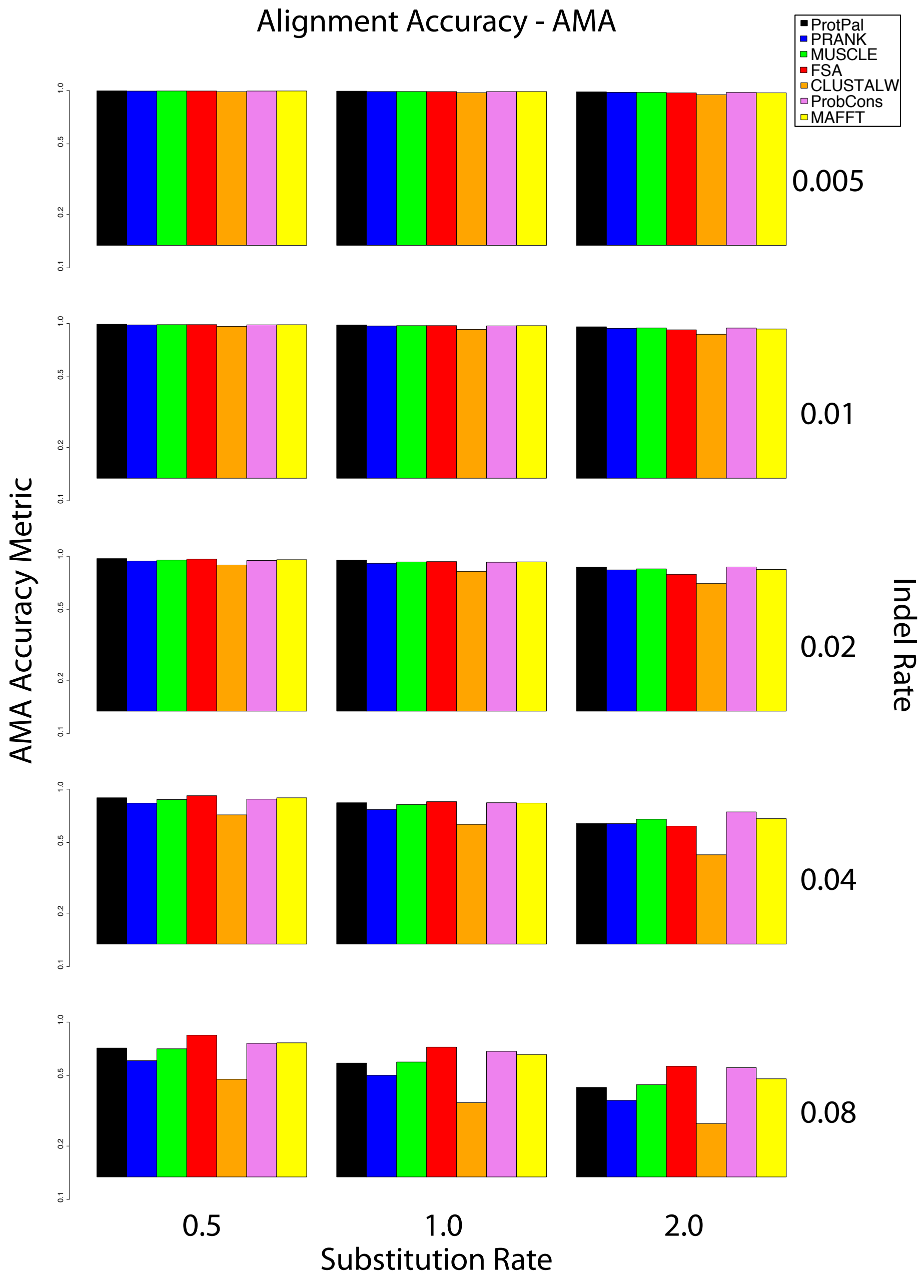

Figure 2: Cross-comparison of AMA scores and rate estimation accuracy reveals that using a single metric to assess alignment accuracy can be unreliable. AMA scores were computed for each programs alignment of only leaf sequences using **cmpalign** from the DART package [15]. AMA scores are comparable across programs until higher indel rates, where FSA performs best—contrasting with Figures 1 and 2 (main text). MUSCLE's accurate deletion rate measurements at high rates and the low corresponding AMA scores suggest a "cancellation of biases".

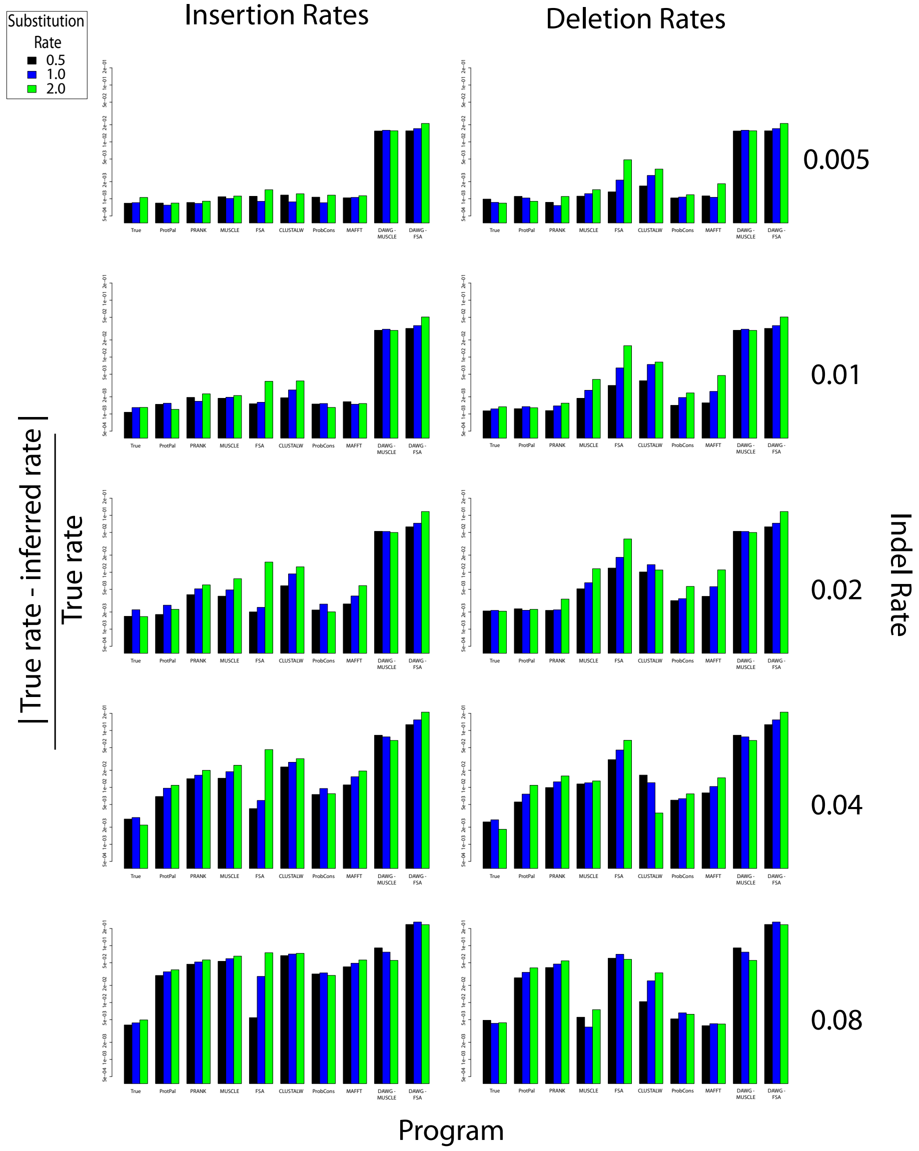

Figure 3: Most programs are relatively robust to variations in the simulated substitution rate, as evidenced by the benchmark data grouped according to substitution rate. Accuracy of rate estimation is plotted as $|true - inferred|$ on the y-axis, with bars grouped by program for each indel rate and 3-tuple of substitution rates. Higher substitution rates often lead to higher error, presumably because they obscure homologies, making it more difficult to distinguish substitutions from indels. FSA appears more sensitive to increased substitutions than other programs - at indel rate 0.02, FSA's insertion rates are as accurate as ProtPal's at 0.5 and 1.0 substitutions per site, whereas at the highest substitution rate (2.0), its error exceeds that of CLUSTALW.

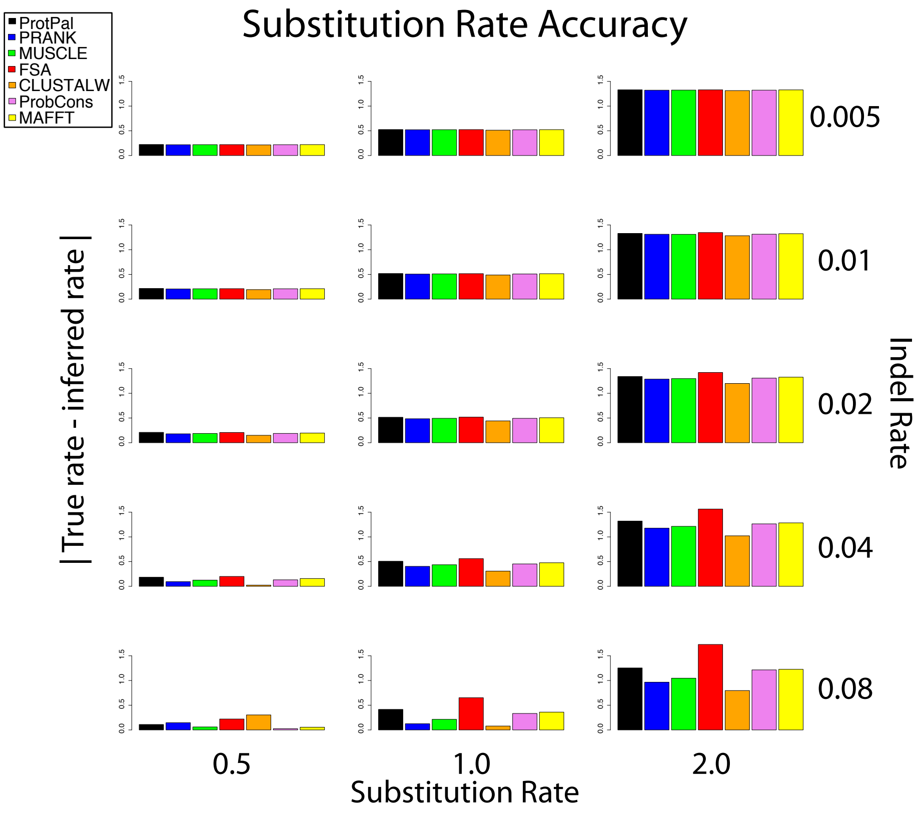

Figure 4: Substitution rates estimated from multiple alignments display comparable accuracy across methods.

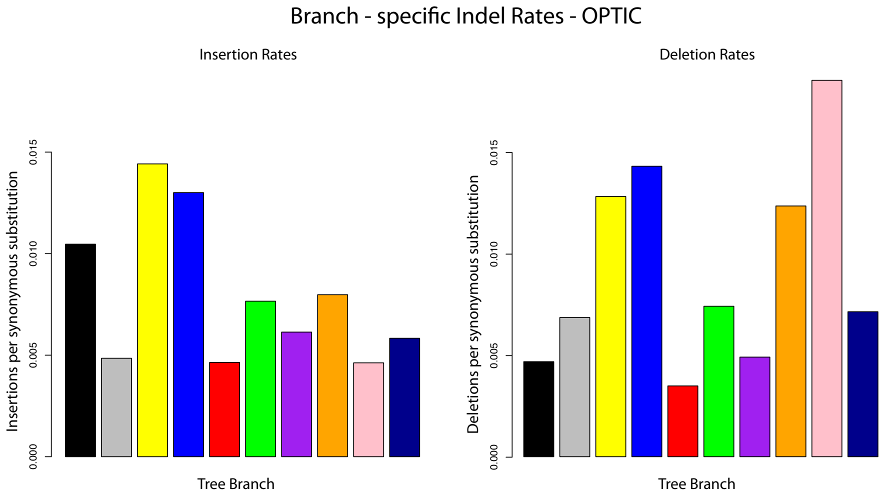

Figure 5: Reconstruction allows for estimation of branch-specific indel rates, revealing possibly interesting signals of evolution. Indel rates were averaged over all alignments, using the species tree shown in Figure 6. The human branch (*Euarchontoglires* - H.*sapiens*) appears to have experienced unusually many insertions. The *Amniota* - *Australophenids* (pink) branch has a higher deletion than insertion rate, though it is difficult to distinguish an insertion on this branch from a deletion on the *Amniota* - G.*gallus* (navy) branch. All other branches are comparable between insertions and deletions. Each bar is colored according to branches in Figure 6.

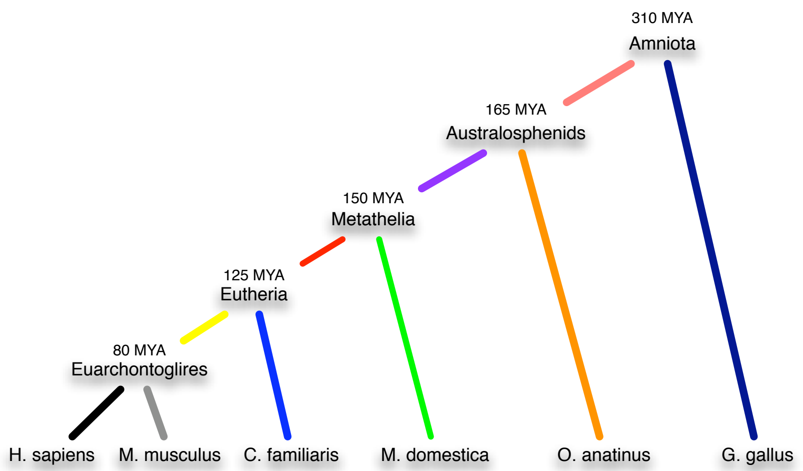

Figure 6: The phylogenetic tree used for analysis of OPTIC data, colored to inform the branch-specific Figure 5.

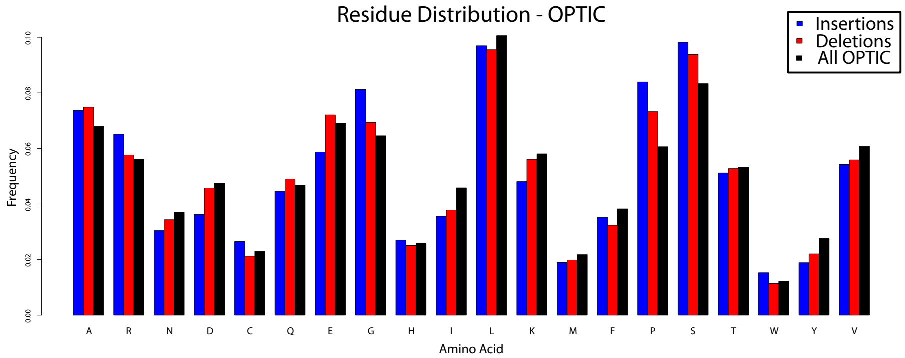

Figure 7: Distributions over amino acids are highly non-uniform, and differ between insertions, deletions, and the overall distribution seen in OPTIC. Inserted, deleted, and all sequences were separately pooled across all OPTIC genes reconstructed and amino acid distributions were computed for each.

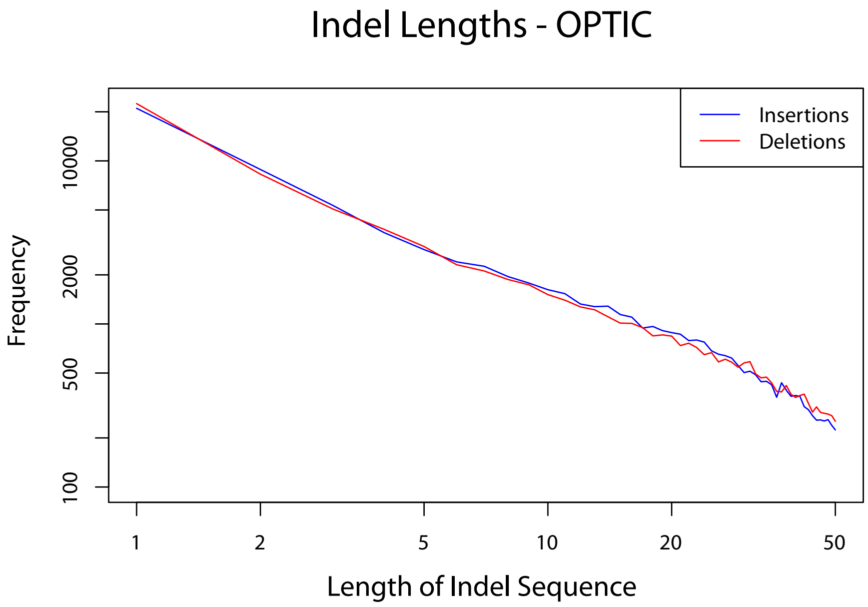

Figure 8: Lengths of inserted and deleted sequences are similarly distributed, in contrast to the conclusions of previous studies, such as [16], which found that deletions were longer relative to insertions in C. *elegans* sequence data. While this may represent a genuine difference in the evolution of human and worm genomes, it is likely that the use of deletion-biased aligners (MUSCLE and CLUSTALW) affected their conclusions.

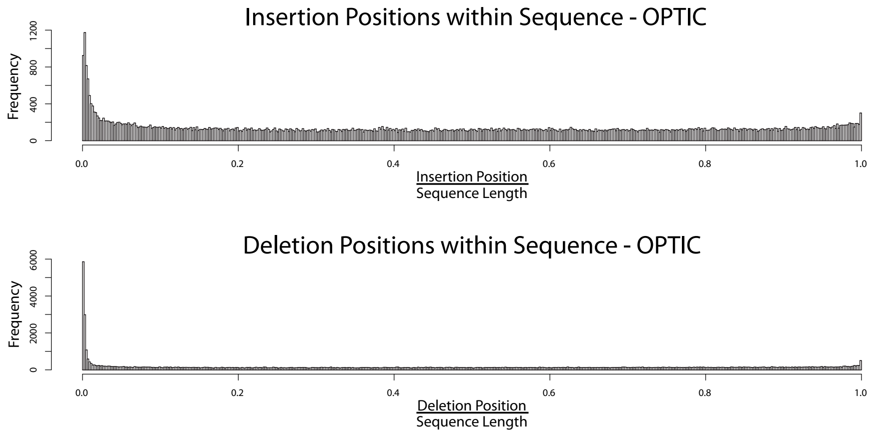

Figure 9: Indels are highly non-uniform in their distribution across genes: we see a 6-fold enrichment for insertions within the N-terminal 1% of the protein sequence, and a 1.4-fold increase within the C-terminal 1%. There is an 14-fold enrichment in deletions within the N-terminal 1% of the protein sequence, and a 1.8-fold increase within the C-terminal 1%. Indel locations are normalized by gene length to enable combining data across all OPTIC genes analyzed. This may be a mix of genuine biology (e.g. indels occur more often near the ends of genes) and artifacts (annotation errors are more likely to occur at the ends of genes).

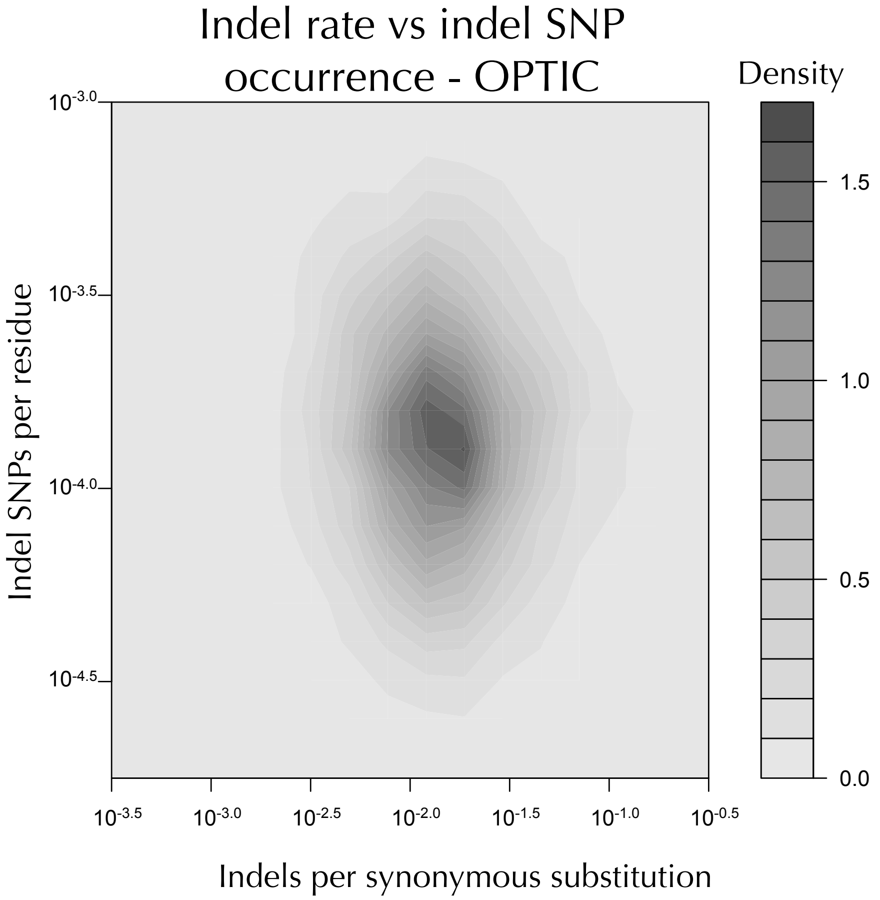

Figure 10: Visualizing the number of indel SNPs per residue (using only human sequence) against the evolutionary indel rate (computed across the *Amniote* clade) shows no correlation.
